# Isolation and characterization of novel bacterial strains exhibiting ligninolytic potential

**DOI:** 10.1186/1472-6750-11-94

**Published:** 2011-10-13

**Authors:** Luaine Bandounas, Nick JP Wierckx, Johannes H de Winde, Harald J Ruijssenaars

**Affiliations:** 1 B-Basic, Julianalaan 67, 2628 BC Delft, The Netherlands; 2 Delft University of Technology, Department of Biotechnology, Julianalaan 67, 2628 BC Delft, The Netherlands; 3 Kluyver Centre for Genomics of Industrial Fermentation, Julianalaan 67, 2628 BC Delft, The Netherlands; 4 BIRD Engineering BV, Westfrankelandsedijk 1, 3115 HG Schiedam, The Netherlands; 5 RWTH Aachen University, Institute of Applied Microbiology, Worringerweg 1, 52056 Aachen, Germany

## Abstract

**Background:**

To expand on the range of products which can be obtained from lignocellulosic biomass, the lignin component should be utilized as feedstock for value-added chemicals such as substituted aromatics, instead of being incinerated for heat and energy. Enzymes could provide an effective means for lignin depolymerization into products of interest. In this study, soil bacteria were isolated by enrichment on Kraft lignin and evaluated for their ligninolytic potential as a source of novel enzymes for waste lignin valorization.

**Results:**

Based on 16S rRNA gene sequencing and phenotypic characterization, the organisms were identified as *Pandoraea norimbergensis *LD001, *Pseudomonas *sp LD002 and *Bacillus *sp LD003. The ligninolytic capability of each of these isolates was assessed by growth on high-molecular weight and low-molecular weight lignin fractions, utilization of lignin-associated aromatic monomers and degradation of ligninolytic indicator dyes. *Pandoraea norimbergensis *LD001 and *Pseudomonas *sp. LD002 exhibited best growth on lignin fractions, but limited dye-decolourizing capacity. *Bacillus *sp. LD003, however, showed least efficient growth on lignin fractions but extensive dye-decolourizing capacity, with a particular preference for the recalcitrant phenothiazine dye class (Azure B, Methylene Blue and Toluidene Blue O).

**Conclusions:**

*Bacillus *sp. LD003 was selected as a promising source of novel types of ligninolytic enzymes. Our observations suggested that lignin mineralization and depolymerization are separate events which place additional challenges on the screening of ligninolytic microorganisms for specific ligninolytic enzymes.

## Background

Lignin is a complex, three-dimensional aromatic polymer consisting of dimethoxylated, monomethoxylated and non-methoxylated phenylpropanoid subunits [1]. It is found in the secondary cell wall of plants, where it fills the spaces between the cellulose, hemicellulose and pectin components, making the cell wall more rigid and hydrophobic. Lignin provides plants with compressive strength and protection from pathogens [2,3]. Presently, millions of tons of lignin and lignin-related compounds are produced as waste effluent from the pulping and paper industries [4]. These amounts are expected to further increase in the near future as a result of the recent developments aimed at replacing fossil feedstocks with lignocellulosic biomass for the production of fuels and chemicals. Generally, biorefinery processes only employ the (hemi-) cellulosic part; the lignin component remains as a low-value waste stream [5] that is commonly incinerated to generate heat and power [6-8]. To date, less than 100 000 t a^-1 ^of lignin obtained from the Kraft pulping process is commercially exploited [9].

Much more value may be obtained if lignin could be utilized as feedstock for value-added chemicals such as substituted aromatics [7,8]. Such valorization would require controlled depolymerization of lignin, which is hampered by its high resistance towards chemical and biological degradation [1]. Lignin can be depolymerized by thermochemical methods such as pyrolysis (thermolysis), chemical oxidation, hydrogenolysis, gasification, and hydrolysis under supercritical conditions [10]. However, many of these processes are environmentally harsh and occur under severe conditions requiring large amounts of energy [11], therefore these processes are not adequate for efficient lignin valorization.

Enzymes could provide a more specific and effective alternative for lignin depolymerization. Furthermore, biocatalytic processes generally take place under mild conditions, which lowers the energy input and reduces the environmental impact [12,13]. A complicating factor for biocatalytic lignin degradation, however, are the structural modifications that lignin undergoes during lignocellulose processing [14,15]. Thus, "industrial" waste lignin may differ considerably from natural lignin, and "natural" ligninolytic enzyme systems may not be the most effective for controlled depolymerization of industrial lignin waste.

The white rot basidiomycetes are the most extensively studied natural lignin degrading microorganisms [16]. These fungi produce an array of powerful ligninolytic enzymes such as laccases, lignin peroxidases (LiP's) and manganese peroxidases (MnP's) [17,18]. These oxidative enzyme systems commonly require low-molecular weight co-factors and mediators, such as manganese, organic acids, veratryl alcohol and substituted aromatics (*e.g*. 4-hydroxybenzyl alcohol, aniline, 4-hydroxybenzioc acid) [12,19]. These mediators are the actual oxidants responsible for lignin degradation, and can penetrate deeply into the lignocellulosic matrix thanks to their limited size. Fungal lignin depolymerization usually results in a variety of low molecular weight aromatic compounds such as guaiacol, coniferyl alcohol, *p*-coumarate, ferulate, protocatechuate, *p*-hydroxybenzoate and vanillate [20,21].

Ligninolytic bacteria are less well studied, but several examples have been found among α-proteobacteria (*e.g*., *Sphingomonas *sp. [22-24]), γ-proteobacteria (*e.g*., *Pseudomonas *sp. [25],) and actinomycetes (*Rhodococcus*, Nocardia and *Streptomyces *sp. [26,27]). The enzymes reported to be involved in bacterial lignin degradation are laccases, glutathione S-transferases, ring cleaving dioxygenases [23,28], monooxygenases and phenol oxidases [29]. Such enzymes are also involved in degradation of polycyclic aromatic hydrocarbons (PAHs), which show similar structural properties and resistance to microbial degradation as lignin [28,30].

Thus, the bacterial ligninolytic potential is still largely unexplored and many novel ligninolytic enzymes may await discovery. These bacterial enzymes may be superior to their fungal counterparts with regard to specificity, thermostability and mediator dependency [2,21,31]. They may also have specific advantages for the depolymerization of the modified lignin residues typically encountered in waste streams from the pulping or 2^nd ^generation biofuel/biobased chemicals industry. In the present study, we describe the isolation and identification of three novel ligninolytic bacterial strains, using a model industrial lignin residue from the Kraft process, which at present is the predominant process in the pulping industry.

## Results

### Enrichment and identification of novel lignin-utilizing bacterial strains

Lignin-degrading microorganisms were enriched in liquid medium with undialysed Kraft lignin (see Methods) as the sole carbon source, using a suspension of soil from beneath rotting logs as the inoculum. After seven successive transfers to fresh lignin medium, individual colonies were obtained on LB agar plates. Seven individual colonies were selected and tentatively identified by partial 16S rRNA gene sequencing. The isolated colonies represented three different species: *Pandoraea norimbergensis*, *Pseudomonas *sp. and *Bacillus *sp. (Table 1). In addition, various standard biochemical tests and cellular fatty acids analysis was performed by the German Resource Centre for Biological Material (DSMZ, Braunschweig, Germany; http://www.dsmz.de/). Additional file 1, Table S1 presents general phenotypic properties of the three isolates and their ability to utilize a selection of substrates.

**Table 1:** Preliminary identification of isolated strains from enrichment cultures by 16S rRNA gene sequencing.

**Isolate name**^**a**^	Accession number (16S rRNA gene sequence)	**Most probable BLAST**^**b **^**hits with 16S rRNA gene**	% Sequence identity	Number of colonies isolated
*Pandoraea norimbergensis *LD001	[Genbank:HQ713574]	*Pandoraea norimbergensis *[Genbank:AF139171.1] [68]	99%	1
*Pseudomonas *sp. LD002	[Genbank:HQ713573]	*Pseudomonas *sp. NZ099 [Genbank:AF388207.1] [69]	99%	4
		*Pseudomonas jessinii *PS06 [Genbank:AY206685.1] [70]	99%	
*Bacillus *sp. LD003	[Genbank:HQ713575]	*Bacillus thuringiensis *CMG 861 [Genbank:EU697392.1] [71]	99%	2
		*Bacillus *sp. NS-4 [Genbank:EU622630.1] [72]	99%	
		*Bacillus cereus *C10-1 [Genbank:AB244465.1] [73]	99%	

^a ^All strains were submitted to the German Resource Centre for Biological Material (DSMZ, Braunschweig, Germany), see Methods for accession numbers.

^ b ^Reference: [67]

### Isolate LD001 - *Pandoraea norimbergensis*

Isolate LD001 was putatively identified as *Pandoraea norimbergensis *by 16S rRNA gene sequencing. Similarities to other members of this genus were lower. The identity of this strain was confirmed by cellular fatty acid profiles (Additional file 2, Table S2) which were typical for the genus *Burkholderia *and related genera such as *Pandoraea*. The general strain characteristics are stated in Additional file 1, Table S1. Interestingly, this strain was unable to metabolize a variety of sugars such as glucose, mannose, arabinose, ribose, maltose, trehalose or cellobiose. It was, however, capable of utilizing fructose as well as a variety of organic acids (citrate, malate, gluconate, mesaconate).

### Isolate LD002 - *Pseudomonas *sp

The partial 16S rRNA gene sequence of isolate LD002 showed highest similarity to *Pseudomonas *sp. NZ099 and *Pseudomonas jessenii *PS06 (Table 1). The cellular fatty acid profile was typical for the genus *Pseudomonas *(Additional file 2, Table S2). Due to the high similarities between different species in this group, both genetically and physiologically, it was not possible to identify this isolate LD002 to the species level. The general characteristics of isolate LD002, designated as *Pseudomonas *sp. LD002, are stated in Additional file 1, Table S1.

### Isolate LD003 - *Bacillus *sp

The partial 16S rRNA gene sequence of isolate LD003 showed 99% similarity to various members of the *Bacillus *genus, such as *B. cereus *and *B. thuringiensis*. The fatty acids profile (Additional file 2, Table S2) of isolate LD003 was typical for that of the *Bacillus cereus *group, which includes *B. cereus, B. thuringiensis *and *B. anthracis*. Due to the high degree of biochemical and morphological similarity between these species, positive species differentiation is difficult [32]. However, *B. anthracis *could be excluded since isolate LD003 exhibited motility, hemolysis and growth on penicillin, which is uncharacteristic of *B. anthracis *[33]. *B. thuringiensis *has commonly been differentiated from *B. cereus *through the presence of plasmid-associated genes that encode insecticidal toxins. These are usually visible as parasporal crystals. However, as such plasmids may be lost or transferred horizontally between the two species, *B. thuringiensis *and *B. cereus *are basically indistinguishable [34]. Thus, although no parasporal crystals were observed with isolate LD003, no definitive identification as either *B. thuringiensis *or *B. cereus *was made, and the isolate was designated *Bacillus *sp. LD003.

### Lignin degrading capacities of the bacterial isolates

The ligninolytic potential of the isolated strains was assessed by their ability to utilize the LMW and HMW fractions of Kraft lignin. The ability to utilize aromatic lignin-monomers as sole carbon source was assessed. In addition, their capacity to decolourize ligninolytic indicator dyes was determined, which is a common method to demonstrate lignin-degrading ability in fungi [35,36].

#### Growth on lignin

Mineral salts medium (MM) containing either the HMW or LMW lignin fraction and additional supplements (see Methods) were inoculated with washed cells from overnight cultures of the three bacterial isolates. The inoculum density was kept between 10,000 and 100,000 CFUs/ml and growth was monitored daily by CFU counts (see Methods). If growth was observed, 1% (v/v) of the culture was transferred to fresh medium to verify that growth occurred on the lignin fractions and not as result of carry-over of medium components that may have escaped the washing step.

**Figure 1:**
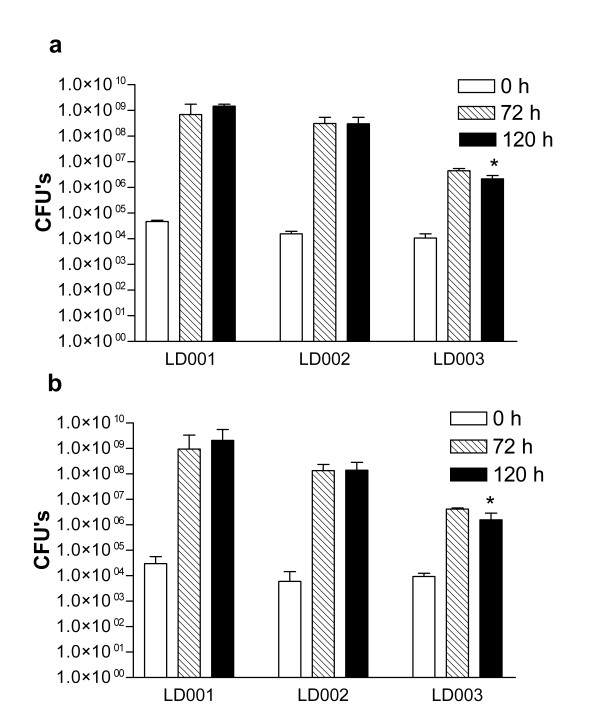
**Growth of bacterial isolates on lignin**. Growth of *P. norimbergensis *LD001, *Pseudomonas *sp. LD002 and *Bacillus *sp. LD003 on a) HMW lignin fraction and b) LMW lignin fraction. Experiments were performed in 4-fold; mean values of CFUs are shown with error bars indicating the maximum deviation from the mean. * Last CFU count for *Bacillus *sp. LD003 was performed at 96 h instead of 120 h.

All the bacterial isolates showed an obvious increase of the number of CFUs in the lignin media (Figure 1). *Pseudomonas *sp. LD002 and *P. norimbergensis *LD001 showed most extensive growth, whereas the CFU increase for *Bacillus *sp. LD003 was relatively modest. Furthermore, *Bacillus *sp. LD003 required supplementation of CuSO_4 _and yeast extract for growth on lignin; without these components, no growth at all was observed (not shown). For all three isolates, the observed growth was similar on the LMW and HMW lignin fractions.

#### Utilization of aromatic monomers

Lignin degrading capacity does not necessarily correlate with efficient growth on lignin, as the released lignin degradation products may not be efficiently metabolized. Particularly actinomycetes have been reported to solubilize and modify lignin, despite exhibiting a limited ability to mineralize lignin [37]. Similarly, the relatively inefficient growth on lignin by *Bacillus *sp. LD003 may indicate a low capacity to depolymerize lignin, but alternatively a low capacity to utilize lignin degradation products. Therefore, the isolated strains were assessed for the capability to utilize monomeric aromatic compounds that are typically associated with lignin degradation.

The spectrum of lignin monomers that could be utilized for growth was relatively limited for all isolates, although *P. norimbergensis *LD001 appeared to have a slightly broader substrate range (Table 2). Remarkably, the alcoholic forms of the aromatic monomers (4-hydroxybenzyl alcohol, vanillyl alcohol, veratryl alcohol, syringol, guaiacol) were not metabolized by any of the isolates. Also the aromatic aldehydes were utilized to a limited extent: vanillin was not degraded by *Pseudomonas *sp. LD002 and syringaldehyde was not utilized by *Bacillus *sp. LD003. In contrast, each isolate consumed all the aromatic acids tested (4-hydroxybenzoic acid, syringic acid and vanillic acid) within 1-2 days. This observation suggests that the isolated strains have a fairly extensive capability for aromatics degradation. However, they appear to lack the alcohol and aldehyde dehydrogenase activities required to oxidize the aromatic alcohols and aldehydes to the carboxylic acid form.

**Table 2:** Growth of bacterial isolates on lignin monomers.

Aromatic compound	*Pandoraea norimbergensis *LD001	*Pseudomonas *sp. LD002	*Bacillus *sp. LD003
phenol	**+**	**+**	**+**
4-hydroxybenzylalcohol	-	-	-
4-hydroxybenzaldehyde	**+**	**+**	**+**
4-hydroxybenzoic acid	**+**	**+**	**+**
guaiacol	-	-	-
vanillyl alcohol	-	-	-
vanillin	**+**	-	**+**
vanillic acid	**+**	**+**	**+**
syringol	-	-	-
syringaldehyde	**+**	**+**	-
syringic acid	**+**	**+**	**+**
veratryl alcohol	-	-	-
anisole	-	-	-

Growth of bacterial isolates in MM with 5 mM lignin monomer as sole carbon and energy source. Growth was considered positive if observed after a successive transfer to fresh medium. Experiments were performed in duplicate. +, Growth; -, no growth

#### Decolourization of ligninolytic indicator dyes

**Figure 2:**
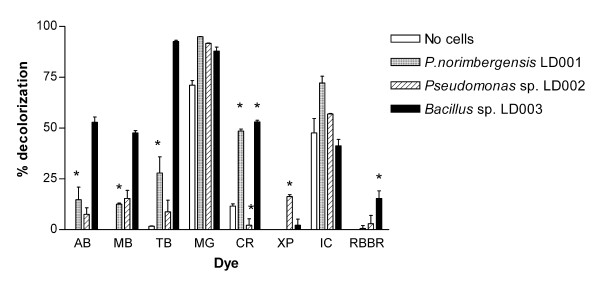
**Decolourization of ligninolytic indicator dyes**. Decolourization (% of initial value) of ligninolytic indicator dyes in LB medium, 25 h after dye addition to exponentially growing cultures of *P. norimbergensis *LD001, *Pseudomonas *sp. LD002 and *Bacillus *sp. LD003. Error bars indicate the maximum deviation from the mean of duplicate experiments. * indicates that dye adsorption to the cell pellet was observed after centrifugation. Dyes: Azure B (AB), Methylene blue (MB), Toluidene Blue O (TB), Malachite Green (MG), Congo red (CR), Xylidine ponceau (XP), Indigo Carmine (IC) and Remazol Brilliant Blue R (RBBR).

**Figure 3:**
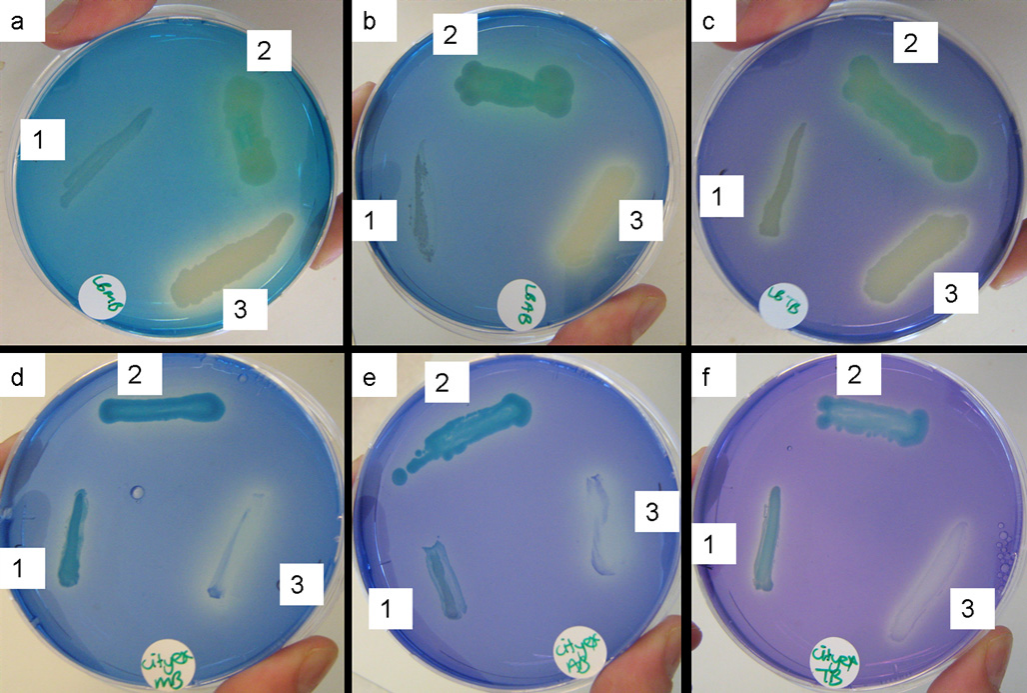
**Decolourization zones in dye-containing plates**. Decolourization zones in dye-containing plates after 72 h of incubation. a) LB with 25 mg/L Methylene Blue (MB); b) LB with 25 mg/L Azure B (AB); c) LB with 25 mg/L Toluidine Blue O (TB); d). MM + 20 mM citrate + 0,5 g/L YE with 25 mg/L Methylene Blue; e) MM + 20 mM citrate + 0,5 g/L YE with 25 mg/L Azure B; f) MM + 20 mM citrate + 0,5 g/L YE with 25 mg/L Toluidine Blue O; 1 - *P. norimbergensis *LD001, 2 - *Pseudomonas *sp. LD002, 3 - *Bacillus *sp. LD003. Experiment performed in duplicate.

As indicated above, growth on polymeric lignin or lignin monomers is not necessarily a good measure of the ligninolytic potential of a bacterial isolate. In order to study ligninolytic potential independently from lignin utilization, the decolourization of synthetic lignin-like dyes may be monitored [38]. This approach was followed for the three isolates, employing a range of lignin-mimicking dyes (Additional file 3, Table S3 for dye structures). Dye decolourization was assessed in liquid assays with growing cultures (Figure 2), as well as in solid phase plate assays (see Figure 3 for an example). Cell pellets and colonies were inspected for dye adsorption and cell-free incubations were assayed as control for abiotic dye decolourization.

*P. norimbergensis *LD001 appeared to decolourize a broad range of dyes in the liquid assays, among which the triarylmethane dye Malachite green (MG) and the indigoid dye Indigo Carmine (IC). However, the abiotic controls of these dyes also showed significant decolourization, indicating that MG and IC were not stable under the conditions tested. Still, *P. norimbergensis *LD001 was found to decolourize IC as well as MG to a larger extent than the abiotic controls (25%, respectively, 24%), suggesting that at least part of the decolourization was biogenic. Upon inspection of the cell pellets Congo Red (CR), Toluidine Blue (TB), Azure B (AB) and Methylene Blue (MB) were found to adsorb to the cells rather than being degraded. Also in plate assays, the decolourization zones for TB and MB were very small and always accompanied by dye adsorption to the colonies (not shown). In contrast to the liquid assays, no decolourization of AB or CR was observed at all in the plate assays. It was therefore concluded that the actual capacity of *P. norimbergensis *LD001 to degrade lignin-mimicking dyes was rather limited, and that the decolourization observed should be attributed mostly to dye adsorption.

*Pseudomonas *sp. LD002 decolourized AB, TB, MB and the azo dye Xylidine Ponceau (XP) by 7-15% in the liquid assays (Figure 2). Of these dyes, only XP was found to adsorb to the cell pellet. Also in the plate assays AB, MB, TB, and XP were decolourized, however, these dyes were also found to adsorb to the colonies (not shown). Therefore, it is unclear to which extent *Pseudomonas *sp. LD002 was truly capable of degrading these dyes, or to which extent decolourization is affected by the growth conditions. IC was not found to be decolourized more than in the abiotic control, whereas MG was decolourized to 21% over the abiotic control.

*Bacillus *sp. LD003 decolourized the thiazine dyes Methylene blue (MB), Azure B (AB) and Toluidene Blue O (TB) to 53, 47 and 8% of their initial value within 25 h in the liquid assays (Figure 2). Similarly, distinct decolourization zones were visible on the solid phase assays within 24 h (Figure 3). Also Remazol Brilliant Blue R (RBBR) appeared to be decolourized by 15% and Congo red (CR) by 52%. However, in solid phase assays, RBBR and CR appeared to adsorb to the cells rather than being degraded. Like *Pseudomonas *sp. LD002, *Bacillus *sp. LD003 did not decolourize IC more than the abiotic control, but MG was decolourized to 18% over the abiotic control. *Bacillus *sp. LD003 did not grow on plates containing MG (not shown), which can probably be attributed to its antimicrobial properties that are particularly effective against Gram-positive microorganisms [39]. As observed for lignin utilization, addition of YE was necessary for dye decolourization by *Bacillus *sp. LD003; however, supplementation with CuSO_4 _could be omitted.

Thus, several lignin-mimicking dyes were decolourized to varying extents by the different bacterial isolates. Especially the dye-decolourizing potential of *Bacillus *sp. LD003 appeared to be substantial, in contrast to its relatively poor growth on lignin (-monomers). This strain decolourized more dyes, and decolourized the dyes more completely, under various conditions, than the other isolates. In order to assess whether the dye-decolourizing activities were extracellular or rather cell-associated, the dyes were also incubated with culture supernatants of *P. norimbergensis *LD001, *Pseudomonas *sp. LD002 and *Bacillus *sp. LD003. These cultures were performed in the presence of lignin or dye to induce the lignin/dye-degrading enzymes. However, none of the culture supernatants showed any dye-degrading capacity, suggesting that the dye-decolourizing activities were cell-associated (not shown).

## Discussion

Three microbial soil inhabitants identified as *Pandoraea norimbergensis *LD001, *Pseudomonas *sp. LD002 and *Bacillus *sp. LD003 were isolated as potential lignin depolymerizing bacteria. The isolated strains showed growth on both high and low-molecular weight lignin fractions, although growth of *Bacillus *sp. LD003 was relatively poor. Typical lignin-associated monomers were utilized to a rather limited extent by all three isolates. Remarkably, the isolated strains appeared to lack the ability to oxidize aromatic alcohols or aldehydes to their corresponding carboxylic acid form.

The ligninolytic potential of the isolates was furthermore assessed by establishing their ability to decolourize synthetic, lignin-like dyes. The recalcitrant thiazine dye Azure B (AB) is particularly suited for this purpose. This dye is decolourized by high redox potential agents, specifically LiP's [17,40,41], whereas it cannot be oxidized by nonperoxidase alcohol oxidases, MnP's or laccases alone [40,42]. In contrast to the other two isolates, *Bacillus *sp. LD003 readily decolourized AB as well as most of the other lignin-mimicking dyes tested. Also other *Bacillus *species as well as members of the *Streptomyces *genus have been reported to degrade AB within 4 - 6 days. These bacteria were isolated from wooden objects, and decolourization of AB was measured to demonstrate lignin peroxidase activity [43]. AB closely resembles methylene blue (MB) and toluidine blue O that were also readily degraded by *Bacillus *sp. LD003. MB has previously been found to be oxidized by lignin peroxidase [44,45].

The seemingly contradictory finding that the highest ligninolytic potential appeared to be associated with the strain that showed poorest growth on lignin may be understood from an ecological perspective. Often, recalcitrant compounds such as lignin are degraded by microbial consortia in which the individual strains have specialized roles: some attack the complex substrate whereas others provide essential nutrients [46]. Ligninolytic bacterial consortia can be found, *e.g*., in the gut of wood-feeding termites[47]. Bacteria like *Rhodococcus erythropolis, Burkholderia *sp., *Citrobacter *sp. and *Pseudomonas *sp. have been isolated from the guts of wood-feeding termites and beetles. These bacteria typically degrade aromatic compounds [25,48,49], which suggests that they feed on the aromatic compounds liberated by the lignin degrading species of the gut microflora. However, lignin-degrading activity has also been reported for certain aromatic compound degraders such as *Pseudomonas *sp. and *Burkholderia *sp. Furthermore, genera such as *Burkholderia, Pseudomonas, Sphingomonas, Bacillus *and *Pandoraea *have been reported to degrade the structurally crucial biphenyl component of lignin, which composes up to 10% of the structure, depending on the lignin type [27,50,51].

Like in other lignin preparations, trace amounts of (hemi)-cellulose may be present in Kraft lignin. This however, is not likely to account for the observed growth on lignin, although the cellulolytic capacity of the isolated strains has not been investigated in detail. Many if not most soil bacteria have incomplete cellulolytic systems [52]. Especially *Pandoraea norimbergensis *is unlikely to utilize cellulose, since it was unable to utilize glucose or cellobiose, both comprising cellulose [53]. Indeed, several *Bacillus sp*. are able to utilize cellulose [54]. The limited growth observed however, on both the high and low molecular weight lignin fractions, in combination with the ability to utilize certain lignin-model dyes clearly indicate the ligninolytic potential of this strain. Other *Bacillus *sp. have accordingly been reported to degrade Kraft lignin [55-57]. In addition, several *Pseudomonas *sp. are able to degrade various lignin preparations such as milled wood lignin, dioxane lignin and lignin from poplar wood [58], further supporting our findings.

In a ligninolytic consortium, *Bacillus *sp. LD003 may fulfill the role of lignin degrader that has to rely on other microbes for specific nutrients, as suggested by its requirement for yeast extract. The other isolates in this study, *Pseudomonas *sp. LD002 and *P. norimbergensis *LD001, showed lesser ligninolytic capacities, but utilized a somewhat wider range of aromatics and did not depend on additional nutrients. Thus, such strains may fulfill the role of nutrient-provider.

The bacterial isolates in this study appear to have an alternative type of ligninolytic system. The enzymes are presumably cell-surface associated, in view of the large size of lignin, whereas fungal lignin degradation occurs via extracellular enzymes and secreted secondary metabolites [59-62]. Thus, a new and presumably vast source may be tapped for novel ligninolytic enzyme activities. A few considerations, however, must be taken into account when hunting for novel ligninolytic activities for lignin valorization. First, the type of lignin to be valorized is a key factor, since the process by which it is obtained will result in structural modifications [15,63,64]. Thus, "natural" ligninolytic systems like those associated with white-rot fungi may not be the most efficient to valorize "industrial" lignin streams such as the Kraft lignin employed in this study. Furthermore, the most efficient lignin mineralizing strains may not be the most efficient lignin depolymerizers. Therefore, lignin degradation should be monitored as directly as possible. Ideally, the actual substrate should be used in degradation assays, but the heterogeneous nature of lignin severely complicates the analytics. Alternatively, synthetic dyes may be used to mimic lignin as we did in the present study. However, the ligninolytic activities obtained by this approach should be evaluated for their utility on the proper type of lignin.

## Conclusions

Microorganisms capable of growing on the complex lignin substrate may be a source of novel enzymes which can be of use for the valorization of waste lignin. Three soil isolates, namely *Pandoraea norimbergensis *LD001, *Pseudomonas *sp. LD002 and *Bacillus *sp. LD003 were identified as potential lignin depolymerizing bacteria, confirming that ligninolytic microorganisms can be found outside the fungal kingdom. All three strains demonstrated growth on both high molecular weight and low-molecular weight lignin fractions, although growth was generally slow and rather poor. The ability to utilize lignin monomers was also relatively limited for all three isolates. The best lignin-like dye decolourizing capacity was observed for the *Bacillus *sp. LD003 and the ligninolytic enzymes and their potential for biocatalytic Kraft lignin depolymerization, are currently under investigation.

## Methods

### Lignin preparation

Commercially available Kraft lignin (Sigma) was used throughout this study. According to the suppliers' specifications, the lignin was water-soluble, contained 4% sulfur impurities, and had an average M_w _of 10.000 Da. Sterile stock solutions of 50 g/L in 15 mM potassium phosphate (KP_i_) buffer (pH 7.6) were prepared by autoclaving.

HPLC analysis of diluted lignin solution (0.5 g/L) showed that low-molecular weight (LMW) aromatic compounds were present (not shown). In order to remove the LMW compounds, a 50-ml aliquot of the lignin stock solution was dialyzed overnight against 500 ml of KP_i _buffer at 2°C using benzoylated dialysis tubing with a 2000-Da MW cut-off (Sigma-Aldrich). The dialysis buffer contained approximately 90% of the LMW lignin fraction, and was stored at -20°C for further testing. Three additional buffer changes (5 L/change) were performed over a 96-h period until no further release of LMW compounds was observed (Additional file 4, Figure S1). The retentate containing the HMW lignin fraction was stored at 2°C until further use.

### Isolation and identification of lignin degrading bacteria

A phosphate buffered mineral salts medium, pH 7, (MM) [65] supplemented with 5 g/L of non-dialysed Kraft lignin, 0.5 mg/L copper sulfate and 0.1 g/L yeast extract (MML), was used for enrichment of lignin-degrading bacteria. As inoculum material, soil collected from beneath decomposing wood logs in a forest near Austerlitz (The Netherlands) was used. The inoculum was prepared by suspending 5 g of soil in 100 ml of sterile 0.9% (w/v) NaCl. After incubating for 1 h at 30°C with shaking at 200 rpm, 5-ml aliquots were used to inoculate four 500-ml Erlenmeyer flasks containing 100 ml of MML. The cultures were grown at 30°C in a shaking incubator and after 48 h, 1-ml aliquots were transferred to fresh MML. Over a period of 24 d, seven successive transfers were performed after which the cultures were streaked onto Luria Broth (LB) agar to obtain pure cultures.

Total DNA was isolated from the pure cultures using a Fast-DNA kit (Q-Biogene). Partial 16S rRNA gene sequences were amplified by polymerase chain reaction (PCR) using primers FD1/2: AGAGTTTGATCMTGGCTCAG and RP1/2: ACGGYTACCTTGTTACGACTT [66] using *Pfu *DNA polymerase (Fermentas). The resulting PCR products were sequenced by MWG Biotech AG and a Basic Local Alignment Search Tool (BLAST) analysis was performed on these sequences to determine the identity of the bacterial isolates [67]. The isolates were further characterized at the German Resource Centre for Biological Material (DSMZ, Braunschweig, Germany), including cellular fatty acid analysis, API, BIOLOG and classical physiological tests. The isolates, *Pandoraea norimbergensis *LD001, *Pseudomonas *sp. LD002 and *Bacillus *sp. LD003 were deposited at the German Resource Centre for Biological Material (DSMZ, Braunschweig, Germany) under the following numbers: [DSMZ: DSM 24563], [DSMZ: DSM 24571] and [DSMZ: DSM 24559], respectively.

### Cultivation of lignin degrading bacteria

The bacteria isolated from the enrichment cultures (*Pandoraea norimbergensis *LD001, *Pseudomonas *sp. LD002 and *Bacillus *sp. LD003) were routinely cultured on Luria broth (LB; 5 g/L NaCl, 10 g/L tryptone, 5 g/L yeast extract, pH 7). Alternatively, the strains were cultured on mineral salts medium, *i.e*., MM supplemented with 40 mM of glycerol, 20 mM of glucose, or 20 mM of citrate.

For monitoring growth on lignin, MM was supplemented with either the LMW or HMW lignin preparations, to a final concentration of 5 g/L. For *Bacillus *sp. LD003, MM was furthermore supplemented with yeast extract (0.1 g/L) and CuSO_4 _(0.5 mg/L). The strains were precultured overnight on mineral salts medium and centrifuged. The cell pellet was washed (in 0.9% NaCl), resuspended, and used to inoculate amber Boston bottles (250 ml) containing 10 ml of lignin medium. The cultures were incubated at 30°C, with shaking, and samples were drawn at regular intervals to monitor growth.

The lignin concentrations employed prevented accurate optical density measurements. Therefore, growth was monitored by colony-forming unit (CFU) counts. For CFU-counts, the cultures were serially diluted in 0.9% (w/v) NaCl and plated on LB agar plates. Daily CFU determinations were made until no further CFU increase occurred. Subsequently, cultures were transferred to fresh media, followed by daily CFU determinations over a period of 5 d (120 h) to exclude that growth was due to the presence of residual carbon from the precultures.

### Dye decolourization assays

Decolourization of lignin-mimicking dyes was assessed both in liquid and in solid-phase assays. The following dyes were selected: Azure B (AB), Indigo Carmine (IC), Malachite Green (MG), Congo Red (CR), Xylidine ponceau (XP), Methylene Blue (MB), Toluidene Blue O (TB) and Remazol Brilliant Blue R (RBBR) (Additional file 3, Table S3). For liquid assays, the individual strains were grown in LB to an OD_600 _of approximately 0.7 - 0.9 (mid-exponential growth). Dyes were added to 25 mg/L (RBBR: 50 mg/L) and cultivation was continued for another 48 h in 250-ml amber Boston bottles at 30°C with shaking at 200 rpm. Cultures without inoculum were included as controls for spontaneous dye decolourization. Samples were drawn at various time intervals and centrifuged for 2.5 min at 15,000 × *g*. The decolourization of a specific dye was calculated as a percentage of the initial absorbance at λ_max _[46]. The colour of the pellet was also visually inspected to establish whether the dye had adsorbed to the cells rather than being degraded.

For solid phase dye decolourization assays, bacteria were streaked onto dye-containing agar plates with various media. Either LB or mineral salts medium (MM) was used, supplemented with a carbon source (glycerol (40 mM) or citrate (20 mM)) and YE (0.5 g/L). Dyes were added to 50 mg/L (IC, MG, CR, XP and RBBR) or 25 mg/L (AB, MB and TB). The plates were monitored daily over a period of 120 h for growth and the development of decolourization zones.

### Analytical methods

For spectrophotometric analysis of the various dyes, a μQuant MQX200 universal microplate spectrophotometer (Bio-tek) was used. The absorbance spectra of the dyes between 200 nm to 800 nm were measured to establish λ_max _for each dye (AB, 650 nm; MB, 665 nm; TB, 635 nm; IC, 615 nm; XP, 505 nm; MG, 615 nm; CR, 470 nm; RBBR, 595 nm). Cell density was measured at 600 nm (OD_600_) using flat-bottom 96-well microplates (Greiner). Cell dry weight (CDW) was calculated from the OD_600 _value. It was established that an OD_600 _of 1 corresponded to 0.42 g/L CDW for the *Bacillus *sp. LB003 and *P. norimbergensis *LB001, respectively, 0.39 g/L CDW for *Pseudomonas *sp LB002. Aromatic compounds were analyzed on an Agilent 1100 HPLC system equipped with a diode array detector set at 254 nm and a 3.5 μM Zorbax SB-C_18 _column (4.6 × 50 mm). 20 mM KH_2_PO_4 _(pH 2) was used as the eluent at a flow of 1.5 ml/min, with a 0 - 20% acetonitrile gradient developing between 0 - 6 min, followed by 20% acetonitrile for a further minute, after which the gradient was decreased to 0% within the final minute.

### Chemicals

All dyes, aromatic compounds and lignin were purchased from Sigma-Aldrich.

## Competing interests

The authors declare that they have no competing interests.

## Authors' contributions

LB participated in the experimental design, performed the experiments and drafted the manuscript. NW participated in the experiments. HJR and JHdW conceived the study, participated in its design and coordination and helped to draft and approve the manuscript. All authors read and approved the final manuscript.

## Supplementary Material

Additional file 1 **Table S1. General characteristics of isolated strains**. Table S1 presents general phenotypic properties of the three isolates and their ability to utilize a selection of substrates. Click here for file

Additional file 2 **Table S2. Fatty acid composition of strains studied**. The identity of the three strains were confirmed by the cellular fatty acid profiles indicated in Table S2. Click here for file

Additional file 3 **Table S3. Dyes used in this study**. The dye structures are represented in Table S3. Click here for file

Additional file 4 **Figure S1. Analysis of LMW and HMW lignin fractions**. a) HPLC analysis of the LMW lignin fraction. Vanillin is indicated as a representative for the aromatic lignin monomers. Other peaks were not identified, but the absorption spectra spectra (not shown) suggested an aromatic structure; b) HPLC analysis of the high molecular weight lignin (HMW) fraction (diluted 10 times prior to HPLC measurement). Less LMW aromatic peaks were observed and vanillin was absent; c) Absorbance spectra of the dialysis buffer between 200 - 400 nm (absorption range for aromatic compounds). The absorption decreased with consecutive buffer changes, indicating that no further LMW aromatic compound were released from the HMW fraction. Click here for file
