# Supplementary material for: Isolation and characterization of novel bacterial strains exhibiting ligninolytic potential
Source: BMC Biotechnol. 2011 Oct 13;11:94. doi: 10.1186/1472-6750-11-94 (PMC3212925; doi:10.1186/1472-6750-11-94)
Supplement: Additional file 1 — Table S1. General characteristics of isolated strains. Table S1 presents general phenotypic properties of the three isolates and their ability to utilize a selection of substrates. [file 1472-6750-11-94-S1.PDF]

Table S1 General characteristics of isolated strains

Strain properties	*Pandoraea norimbergensis* LD001	*Pseudomonas* sp. LD002	*Bacillus* sp. LD003
Shape of cells	Rods	Rods	Rods
Width of cells (μm)	0,7 – 0,8	0,7 – 0,8	0,9 – 1,1
Length of cells (μm)	2,0 – 3,5	2,5 -> 5,0	2,0 – 4,5
Gram reaction	Gram negative	Gram negative	Gram positive
Motility	+	+	+
Oxidase	+	+	+
Catalase	+	+	+
ADH	+	+	ND
Urease	-	-	ND
Lysis by 3% KOH	+	+	ND
Aminopeptidase	+	+	ND
Lecithinase	ND	+	+
Levan from sucrose	ND	+	ND
Indole reaction	ND	ND	-
Phenylalaninedesaminase	ND	ND	-
Arginine dihydrolase	ND	ND	-
Denitrification	-	-	NO_2 from NO_3
Hemolysis	ND	ND	+
Fluorescent pigments	ND	+	ND
VP reaction	ND	ND	+
pH in VP broth	ND	ND	4.9
Anaerobic growth	ND	ND	+
Negative growth	42ºC	41ºC	50ºC
Growth on *Pseudomonas* Isolation Agar (PIA)	Confluent growth	Light growth	ND
Growth in medium pH 5.7	ND	ND	+
Growth in lysozyme broth	ND	ND	+
Growth in 2 – 7% NaCl	ND	ND	+
**Growth on LB agar + antibiotic after 1 day:**			
10 μg/ml tetracycline	+	-	-
30 μg/ml tetracycline	-	-	-
10 μg/ml gentamycin	+	-	-
25 μg/ml gentamycin	+	-	-
100 μg/ml ampicillin	+	+	+
5 μg/ml kanamycin	+	-	+
50 μg/ml kanamycin	+	-	-
5 μg/ml erythromycin	+	+	-
10 μg/ml kanamycin	+	+	-
50 μg/ml kanamycin	+	+	-
10 μg/ml chloramphenicol	+	+	-
100 μg/ml spectinomycin	ND	ND	+

200 μg/ml spectinomycin	ND	ND	-
**Hydrolysis of:**			
gelatine	+	+	+
esculin	-	-	+
casein	-	ND	+
starch	ND	ND	+
Tween 80	ND	ND	-
**Utilization of:**			
glucose	-	+	+
phenylacetate	+	-	ND
citrate	+	+	+
malate	+	+	ND
mannose	-	+	ND
mannitol	-	+	- for acid from D-mannitol
gluconate	+	+	ND
maltose	-	-	ND
trehalose	-	-	ND
citraconate	-	-	ND
adonitol	-	ND	ND
m-hydroxybenzoate	+	ND	ND
arabinose	-	+	- for acid from L-arabinose
mesaconate	+	ND	ND
ribose	-	ND	ND
N-acetylglucosamine	-	ND	ND
cellobiose	-	ND	ND
m-inositol	ND	-	ND
sorbitol	ND	-	ND
erythrite	ND	-	ND
hippurate	ND	-	ND
D-mandelate	ND	-	ND
2-ketogluconate	ND	+	ND
propionate	ND	ND	-
fructose	+	+	+ for acid from D-fructose
D-xylose	ND	ND	- for acid from D-xylose

+,positive; - ,negative; ND, not determined
