# Supplementary material for: Isolation and characterization of novel bacterial strains exhibiting ligninolytic potential
Source: BMC Biotechnol. 2011 Oct 13;11:94. doi: 10.1186/1472-6750-11-94 (PMC3212925; doi:10.1186/1472-6750-11-94)
Supplement: Additional file 2 — Table S2. Fatty acid composition of strains studied. The identity of the three strains were confirmed by the cellular fatty acid profiles indicated in Table S2. [file 1472-6750-11-94-S2.PDF]

Table S2 Fatty acid composition of strains studied

Fatty acids	*Pandoraea norimbergensis* LD001	*Pseudomonas* sp. LD002	*Bacillus* sp. LD003
10:0	trace	ND	ND
10:0 3OH	ND	4.00	ND
12:0 ISO	ND	ND	trace
12:0	2.54	1.24	trace
13:0 ISO	ND	ND	8.47
13:0 ANTEISO	ND	ND	1.53
13:1 AT 12-13	1.48	ND	ND
12:0 2OH	trace	6.20	ND
12:1 3OH	ND	trace	ND
12:0 3OH	ND	4.46	ND
14:0 ISO	ND	ND	5.81
14:1 w5c	trace	ND	ND
14:0	trace	trace	2.37
Unknown 14.502	trace	ND	ND
15:0 ISO	ND	ND	28.23
15:0 ANTEISO	ND	ND	6.13
15:1 w6c	trace	ND	ND
15:0	trace	ND	trace
16:1 w7c alcohol	ND	ND	1.2
16:0 ISO	ND	ND	8.4
16:1 w11c	ND	ND	trace
16:1 w5c	trace	ND	ND
16:0	20.64	32.66	5.54
15:0 2OH	ND	ND	trace
ISO 17:1 w10c	ND	ND	3.08
ISO 17:1 w5c	ND	ND	3.84
ANTEISO 17:1 w9c	trace	ND	ND
17:1 ANTEISO A	ND	ND	1.07
17:0 ISO	ND	ND	7.88
17:0 ANTEISO	ND	ND	1.66
17:0 CYCLO	14.7	12.22	ND
17:0	trace	ND	ND
16:1 2OH	trace	ND	ND
16:0 2OH	trace	ND	ND
16:0 3OH	3.14	ND	ND
18:1 w7c	26.14	11.72	ND
18:1 w5c	trace	ND	ND
18:0	1.01	trace	ND
19:0 CYCLO w8c	7.74	ND	ND
18:1 2OH	1.07	ND	ND
Summed feature 2: 12:0 ALDE, 16:1 ISO I/14:0 3OH	Trace 4.51	ND	3.21
Summed feature 3: 16:1 w7c/15 iso 2OH	13.06	25.96	9.14

Results of fatty acids expressed as percentages. ND: Not detected. Trace: trace amount detected (less than 1 %)
