# Supplementary material for: Isolation and characterization of novel bacterial strains exhibiting ligninolytic potential
Source: BMC Biotechnol. 2011 Oct 13;11:94. doi: 10.1186/1472-6750-11-94 (PMC3212925; doi:10.1186/1472-6750-11-94)
Supplement: Additional file 3 — Table S3. Dyes used in this study. The dye structures are represented in Table S3. [file 1472-6750-11-94-S3.PDF]

Table S3 Dyes used in this study

Dye and dye classification	Structure
Methylene blue (Thiazine)	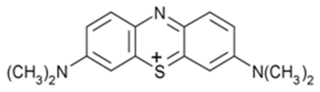
Azure B (Thiazine)	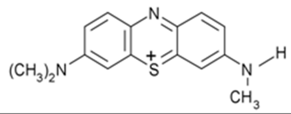
Toluidene Blue O (Thiazine)	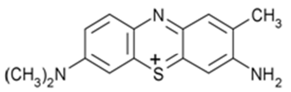
Malachite green (Triarylmethane)	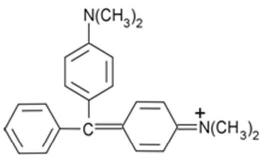
Indigo Carmine (Indigoid)	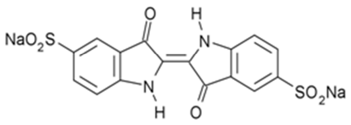
Congo red (Azo)	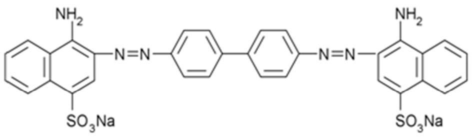
Xylidine ponceau (Azo)	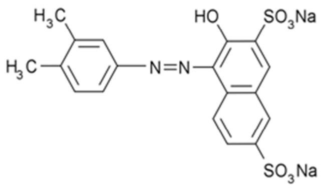
Remazol Brilliant Blue R (Anthraquinone)	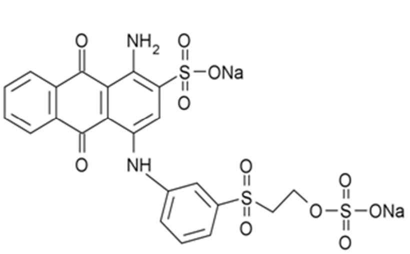
