# Supplementary material for: Isolation and characterization of novel bacterial strains exhibiting ligninolytic potential
Source: BMC Biotechnol. 2011 Oct 13;11:94. doi: 10.1186/1472-6750-11-94 (PMC3212925; doi:10.1186/1472-6750-11-94)
Supplement: Additional file 4 — Figure S1. Analysis of LMW and HMW lignin fractions. a) HPLC analysis of the LMW lignin fraction. Vanillin is indicated as a representative for the aromatic lignin monomers. Other peaks were not identified, but the absorption spectra spectra (not shown) suggested an aromatic structure; b) HPLC analysis of the high molecular weight lignin (HMW) fraction (diluted 10 times prior to HPLC measurement). Less LMW aromatic peaks were observed and vanillin was absent; c) Absorbance spectra of the dialysis buffer between 200 - 400 nm (absorption range for aromatic compounds). The absorption decreased with consecutive buffer changes, indicating that no further LMW aromatic compound were released from the HMW fraction. [file 1472-6750-11-94-S4.PDF]

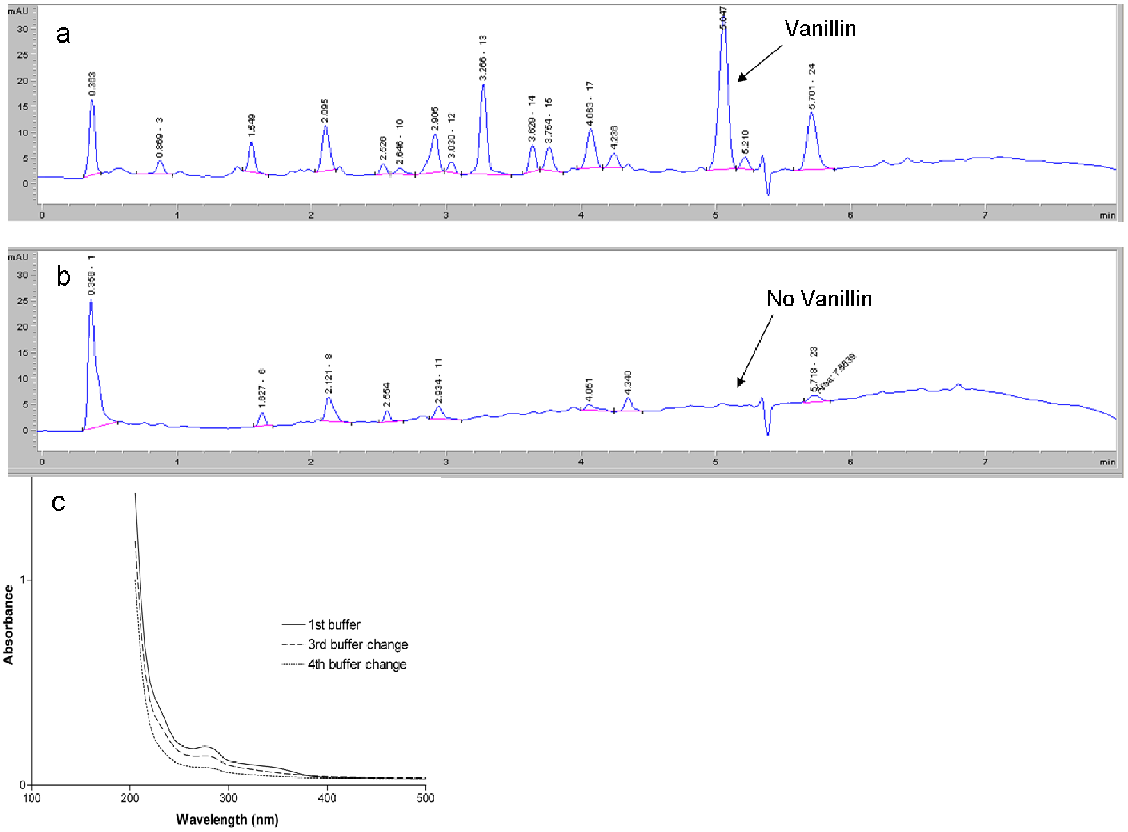

Figure S1 a) HPLC analysis of the LMW lignin fraction. Vanillin is indicated as a representative for the aromatic lignin monomers. Other peaks were not identified, but the absorption spectra (not shown) suggested an aromatic structure; b) HPLC analysis of the high molecular weight lignin (HMW) fraction (diluted 10 times prior to HPLC measurement). Less LMW aromatic peaks were observed and vanillin was absent; c) Absorbance spectra of the dialysis buffer between 200 - 400 nm (absorption range for aromatic compounds). The absorption decreased with consecutive buffer changes, indicating that no further LMW aromatic compound were released from the HMW fraction
